# A pilot study for the prediction of liver function related scores using breath biomarkers and machine learning

**DOI:** 10.1038/s41598-022-05808-5

**Published:** 2022-02-07

**Authors:** Rakesh Kumar Patnaik, Yu-Chen Lin, Ashish Agarwal, Ming-Chih Ho, J. Andrew Yeh

**Affiliations:** 1 Institute of NanoEngineering and MicroSystems, National Tsing Hua University, Hsinchu, 30013 Taiwan; 2 Department of Surgery, National Taiwan University Hospital and College of Medicine, Taipei City, 100225 Taiwan; 3 Department of Surgery, National Taiwan University Hospital Hsin-Chu Branch, Zhubei City, Hsinchu County 100225 Taiwan

**Keywords:** Biomarkers, Diseases, Health care, Health occupations, Medical research, Engineering

## Abstract

Volatile organic compounds (VOCs) present in exhaled breath can help in analysing biochemical processes in the human body. Liver diseases can be traced using VOCs as biomarkers for physiological and pathophysiological conditions. In this work, we propose non-invasive and quick breath monitoring approach for early detection and progress monitoring of liver diseases using Isoprene, Limonene, and Dimethyl sulphide (DMS) as potential biomarkers. A pilot study is performed to design a dataset that includes the biomarkers concentration analysed from the breath sample before and after study subjects performed an exercise. A machine learning approach is applied for the prediction of scores for liver function diagnosis. Four regression methods are performed to predict the clinical scores using breath biomarkers data as features set by the machine learning techniques. A significant difference was observed for isoprene concentration (*p* < 0.01) and for DMS concentration (*p* < 0.0001) between liver patients and healthy subject’s breath sample. The R-square value between actual clinical score and predicted clinical score is found to be 0.78, 0.82, and 0.85 for CTP score, APRI score, and MELD score, respectively. Our results have shown a promising result with significant different breath profiles between liver patients and healthy volunteers. The use of machine learning for the prediction of scores is found very promising for use of breath biomarkers for liver function diagnosis.

## Introduction

The liver is the primary organ of the body, responsible for metabolism and protein absorption. Many diseases affect the liver, including hepatitis, fibrosis, and cirrhosis. Liver cirrhosis and liver cancer stand at 11th and 16th rank as the most common causes of death globally, with yearly more than 1 million and 0.7 million deaths, respectively^1^.

The gold standard method for identifying the fibrosis stage is liver biopsy, it is an invasive test procedure with around 6% of patients found post complicacy^2^ and bleeding issues^3^. Liver diagnosis is also done using liver function tests and imaging methods have been established to distinguish the degree of fibrosis; though the blood test needs standard laboratories and the imaging techniques need costlier equipment, operator expertise, and other contrast-related issues are inherently associated with this technique^4^.

A liver function test is the first step in diagnosing various liver diseases by measuring certain proteins and liver enzymes in the blood sample. After getting the required data from the clinical parameters from the blood test, it is possible to calculate Child–Pugh (CTP), AST to PLT ratio (APRI), and Model for End-Stage Liver Disease (MELD) clinical scores that help doctors determine the severity of the disease progression and predict the survival rate after disease.

Thousands of VOCs were already identified in the exhaled breath^5^. Many metabolic reasons regulate the VOC concentration or the VOCs profile^6,7^. VOC biomarkers in the breath reflect an enormous number of sicknesses in many research articles^8,9^. Breath is accessible in an adequately limitless stock, which gives more logical benefits than blood test procedures.

In comparison to the blood test, a breath test facilitates a non-invasive measurement where the concentration of target biomarkers reflects the change in body metabolism condition caused by sickness or infection. Breath VOCs concentration profile holds the information about body metabolism that is unique because VOCs in the bloodstream are transferred to the exhaled air and come out. Hence, breath empowers the entire body blood inspection. Any sort of inflammation, infection, presence of tumor cells and oxidative stress cause a change in the concentration of certain compounds found in the exhaled breath ^10–12^.

Isoprene is a by-product of the glycolysis cycle which has been observed in an in-vitro experiment^13^. Isoprene is assumed to be a typical derivative of cholesterol biosynthesis as an outcome of the non-enzymatic degradation of dimethylallyl-pyrophosphate^14–16^, a member of cholesterol and isoprenoids synthetic path-way^17,18^. Even previous studies have shown that the isoprene shows the highest area under the curve for discriminating fibrosis stages as compared to other compounds^19^. One study reveals that the in healthy pupils aged 7 to 18 years range of isoprene is from 20 to 240 ppb^20^. The range of isoprene is broad and is overlapped between healthy and patients.

Another VOC that holds the potential to be a liver breath biomarker is DMS. Breath DMS level increases when a liver deteriorates and leads to cirrhosis condition^21–23^. The liver regulates the synthesis and metabolism of hydrogen sulphide (H_2_S). Methylation of H_2_S produces DMS^24^. There is no clear explanation for the altered regulation of H_2_S metabolism. But studies show that hepatic dysfunction disturbs the process by affecting the enzymes responsible for H_2_S absorption so part of the H_2_S converted to DMS^25^. Liver is responsible for producing P450 metabolic enzymes. A cirrhotic liver got deprived of enough CYP2C19 compounds belongs to P450 enzyme that reduces the digestion of limonene and causes an increase in limonene levels in the breath^26^. Limonene is common to find in a healthy person and liver patient breath because it is commonly found in food and beverages which give citrus fruit flavour, and this is hard to avoid in a regular diet^27^. Limonene is obvious to appear in the exhaled breath of both the healthy and the patients’ group, but the concentration remains higher in the latter group.

There are previous studies that address multiple potential biomarkers related to liver disease. Some studies even emphasize on categorizing patients from healthy subjects using VOCs concentration^28,29^. A diseased-liver alter the regulation of metabolism, so we hypothesized that there will be some makeable changes between these three biomarkers which can be detected by breath analysis. The objective of the study is to use the biomarkers concentration profile to predict the liver-related clinical scores. In this work, a panel of biomarkers is considered which includes isoprene, limonene, and DMS. The designed breath collection protocol includes a brief duration exercise which is a novel approach according to the best of our knowledge in the field of liver-related study. Particularly a previous study indicates isoprene concentration increases after performing an exercise^30^. The change in the concentration profile of isoprene is included as an important parameter.

In the proposed work we not only observe biomarkers concentration but also try to predict scores using breath data and compare it with the real value of scores which are derived from blood test data. Different regression algorithms are employed to observe the predictions on a designed dataset that comprises the said VOC concentration. Further the variation of the trend change in isoprene concentration is studied. Four different regression methods are applied in this work, that are Linear regression (LR), Support Vector Regression (SVR), Random Forest Regression (RFR), and Extra Tree Regression (ETR). The performance of regression analysis is evaluated based on mean absolute error (MAE) and root mean squared error (RMSE) metrics. The overall performance was also discussed using adjusted R-square value and AIC score.

## Methods

### Study subject selection and clinical parameters

The choice of the study population is designed to represent real-world population distribution. The healthy members are chosen based on a preliminary liver donors’ criterion. Based on their blood test result, age and BMI those individuals were considered in the study who have a perfect liver. In our recent work, it was described that ten years of a survey of 473 liver donors morbidity after liver hepatectomy^31^. This study describes that the average age of liver donors is not more than 30 years. For this reason, in this study, the healthy group members are younger than the liver patients.

Particularly aging has no significant effect on the chosen potential VOCs concentration if exists than at very old age^30,32^. The literature describes there is no indication of significant change in isoprene concentration with gender and age. Some studies found that within the adult age group, there is little evidence on breath isoprene concentration varies with aging. The second biomarker, limonene is digested by liver P450 enzymes. Studies show that there is an effect of aging on the production of P450 metabolizing enzymes after 70 years and some coenzymes of P450 family production remain unchanged from 23 to 80 years^33,34^. Another in-vitro study shows that in rat liver, the decrease P450 enzymes shown in aged group of samples^35^. The metabolizing P450 enzyme which is responsible for the digestion of limonene doesn’t vary with age and only liver disease is responsible for an increase in the concentration. The effect of aging on DMS concentration is not commonly found. But a study shows the effect of aging on rat liver and the regulation of sulfur compounds. This study concludes that there is a mixed trend observed in the production of sulfur compounds with aging^36^.

The breath test protocol and research method ethics were approved by the Institutional Review Board of NTUH, Taipei, Taiwan (REC: 201912138RINB). All research was performed according to the ethics guidelines and a signed informed consent form was obtained from all study participants. The breath test was done in NTUH, Taiwan. In the inclusion criteria, the allowed age limit is 20–70 years. Study subjects need to agree to perform staircase walking for two minutes and their heart rate must reach a minimum of 100 bpm. All study subjects need to maintain a minimum of 8 h of fasting before the breath test. Study subjects need to avoid smoking and alcoholic drink for 24 h before the breath test. The exclusion criteria are that healthy subjects must not have past or present liver ailment issues and lungs disease. Patients are advised to withdraw if they feel any discomfort while following the breath protocol. Seventeen liver patients were enrolled in this study at NTUH, Taipei, Taiwan. Total seventeen patients that include eight Patients who had hepatic cellular carcinoma issues, six patients admitted for surgery, two patients joined for the treatment of cholangiocarcinoma and four out of the considered patient list came for transplant evaluation. Twenty-eight healthy volunteers were recruited as a healthy group and a liver function test is conducted to make sure no present symptoms or indication of the ill liver. Eight different common protein and liver enzymes that are commonly monitored in liver patients admitted in NTUH, Taipei considered in our study. This includes albumin (ALB), total bilirubin (TBIL), Aspartate transaminase (AST), Alanine transaminase (ALT), platelet count (PLT), prothrombin time (PT), normalized prothrombin time (INR), and creatinine (CRE). Table 1 describes the mean and standard deviation of said liver-related protein and enzymes. Other than this information about hepatic encephalopathy (HE), ascites (ASC), serum sodium (Na), dialysis information (DLYS) was also retrieved. Three clinical scores formula and calculation methods are shown in supplementary documentation (Section 1: Clinical score calculation). The following table explains all the relevant information.

**Table 1:** Demographic and clinical parameters of study subjects (mean ± SD).

Parameters	Healthy (n = 28)	Liver patient (n = 17)	Total (45)
Age (years)	24.78 ± 5.07	59.76 ± 7.31	38 ± 18.15
Height (cm)	168.03 ± 8.75	163.02 ± 7.6	166.14 ± 8.6
Weight (kg)	63.85 ± 9.87	69.18 ± 13.14	65.86 ± 11.38
Albumin (g/dL)	4.64 ± 0.36	4.1 ± 0.69	4.4 ± 0.57
T-bilirubin (mg/dL)	0.74 ± 0.29	1.25 ± 0.95	0.93 ± 0.66
AST (units/L)	18.85 ± 4.64	46.23 ± 20.04	29.2 ± 18.42
ALT (units/L)	15.64 ± 9.17	44.23 ± 28.73	26.44 ± 23.41
Platelet count	252.17 ± 57.51	156.41 ± 97.83	216 ± 87.83
Prothrombin time (PT) (sec.)	10.91 ± 0.48	11.3 ± 0.81	11.06 ± 0.64
Normalized PT	1.05 ± 0.04	1.07 ± 0.07	1.06 ± 0.06
Creatinine (mg/dL)	0.84 ± 0.17	0.81 ± 0.15	0.83 ± 0.16

### Breath collection protocol

Before beginning the breath collection one prepared questionnaire is answered by the study subjects. The questionnaire gives us information about their brushing teeth, fasting, and cigarette smoking. To avoid the collection of target VOCs which may pre-exist in the mouth, rinsing the mouth before collection is essential.

One liter gas sampling aluminum bag is used for breath collection. A commercial breath collection mask has not been used to avoid any kind of discomfort to the patients. All samples were collected in a new bag. Breath samples were collected in three different bags in different situations. For the first bag of breath collection, study subjects were asked to first slow down their breathing until their heart rate reach 60–80 bpm. After the heart rate remains stable for about one minute, subjects are asked to blow into the bag (bag 1). The second bag (bag 2) is collected immediately after the subject performs staircase walking for a minimum of two minutes and the heart rate shoots up at least 100 bpm. The third bag is collected after 15 min of rest and after observing the heart rate is stable between 60 and 80 bpm (bag 3). After the breath is collected in the bag within one-hour sampling process starts. Bag 1 and bag 3 is collected at rest for that reason the heart rate is expected to be at normal reading. The exercise section is systemized using 2 min of staircase walking after the heart rate increases to 100 bpm on the same facility.

### Breath analysis

**Figure 1:**
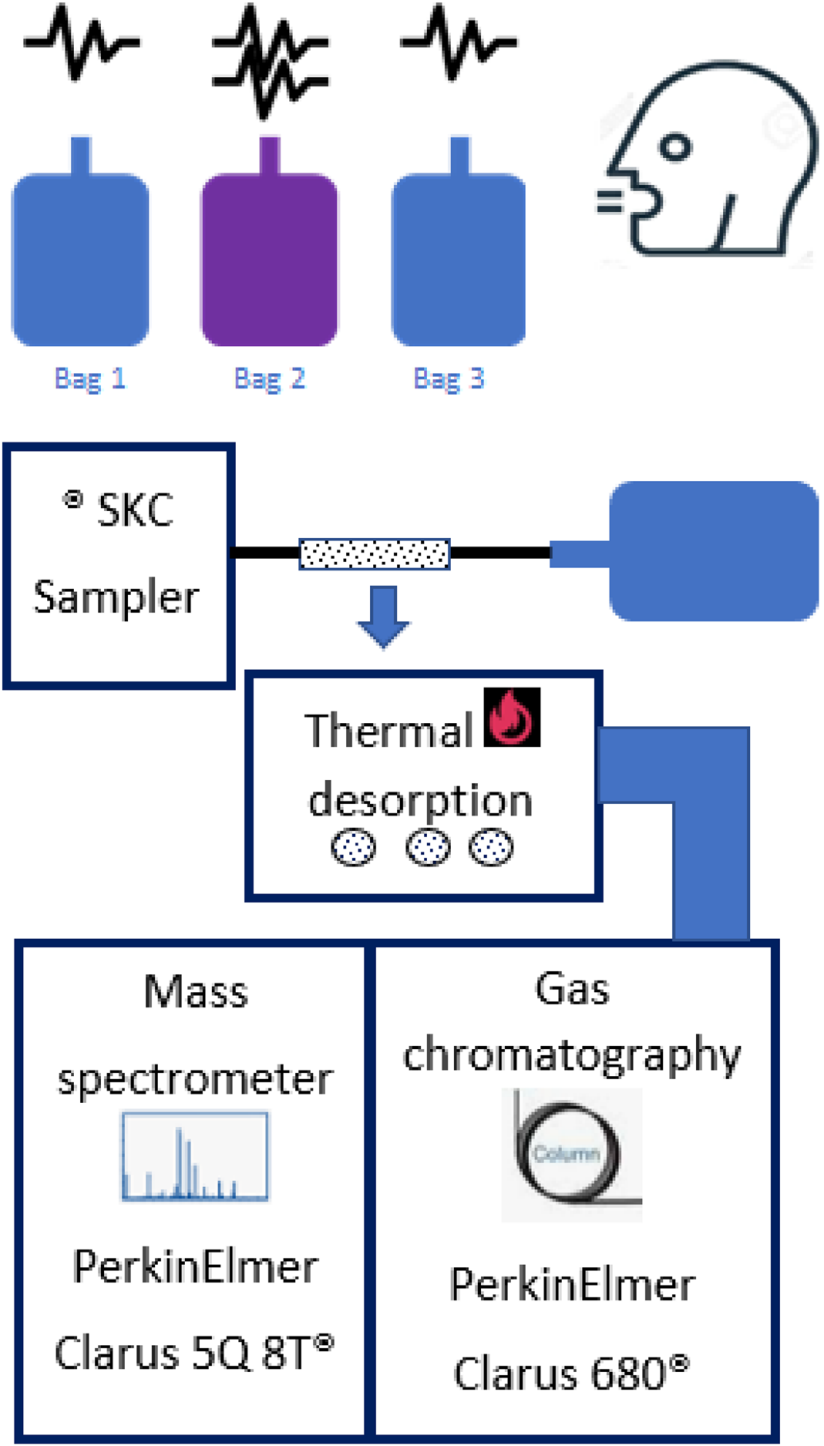
The layout of breath collection and analysis setup.

Breath samples are analysed using a GCMS-Clarus 680 instrument (PerkinElmer ®, USA). At first samples from the bags were sampled into the ‘Carbotrap’ desorption tubes using a pump (SKC®) and then an Automatic Thermal Desorption (ATD) unit (TurboMatrix 350 PerkinElmer®, USA) that desorb the sample from the tube into the GC column. ATD comes with an automatic sample tube handling unit. That selects one tube at a time and heats the tube. Then the sample was transferred into a Trap unit then transferred to the GC column^37^. A conceptual experimental setup diagram is shown in Fig. 1.

The VOCs concentration was calculated utilizing a GCMS attached with a programmed desorption unit. The ATD has a desorption unit and a facility for the build-up of low-concentrate VOCs. The GC/MS station utilized helium gas of 99.9995% pure as the transporter gas. The GC section was an Elite 624 polar column (PerkinElmer, USA) with length, inside breadth, and film thickness is 60 m, 0.25 mm and 1.4 µm respectively. This GC has a single section. The temperature ramp-up of the profile has three stages. At first, it is 35-°C for 5 min. At that point at a pace of 7-°C/min, it expanded up to 90 °C and afterward, the rate expanded to 30-°C/min until it arrived at 220-°C. The temperature needs to rise gradually so the peaks show up at some point and a moderate temperature ramp-up profile keeps up to focus for those mixes with less boiling point. Abrupt increment in column temperature cause short retention time and generally the peaks gets overlapped. The mass-to-charge proportion (m/z) was considered between the range of 20 and 300. VOCs were distinguished utilizing the mass information base designed by NIST provided by GC–MS software. Quantification is done by peak area comparison with reference to the calibration done using the standard compounds.

## Regression analysis

### Dataset

Three different datasets are prepared as CTP score, APRI score, and MELD score as output variable, respectively. There are seven breath parameters as input features kept constant in all three datasets. Those features are isoprene concentration from three different bags, isoprene concentration difference from bag2 to bag1 and bag2 to bag 3, mean limonene concentration, and mean DMS concentration. All features are numerical and carry no missing value. The distribution plot for the three clinical scores is shown in supplementary Figs. S1–S3. The machine learning approach we adapted is not to distinguish between patients and healthy people using the dataset. Regression is a special application of classification rules which is used when the value of prediction is based on a dataset rather than mapping the data according to different labels or classes. The regression approach is commonly used to predict continuous quantity, but it can predict the discrete value but in the form of integer quantity^38^. Based on the dataset and objective of the work regression method is chosen over multiclass classification. Classification on this dataset will lead to value error because the least populated class in the target has less than two instances. Which may lead to an unacceptable classification and confusion matrix that will produce an improper F1 score and recall. For this reason, the approach made for prediction using the regression method.

### Grid search and hyper-parameter tuning

Four different regression methods applied in this work, are Linear regression (LR), Support Vector Regression (SVR), Random Forest Regression (RFR), and Extra Tree Regression (ETR). These methods are implemented using ‘Jupyter 6.0.1’ version for Python code development. The simple and the classic method is linear regression is as a baseline regressor. Support Vector Machine which is very popular in creating a hyperplane and forming a decision boundary. Ensembles learning method also applied to the dataset for score prediction.

In this work, grid search is run for SVR, RFR, and ETR algorithms for three different datasets. Each dataset is split into 70% as the training set and 30% as the test set. The test size was kept independent from the training data and unseen to the model. The parameters and their optimum value with scoring (negative mean absolute error) are shown in supplementary Table S2. Using the grid search function, the optimum value was found for hyperparameters of said methods for three different scores. For the SVR model, linear kernel with 0.01 regularization factor for the CTP and MELD whereas for APRI it is 1. The major hyperparameter in the ensemble learning model (ETR and RFR) is the number of trees. For the ETR model the optimum number of trees for CTP is 10 and for APRI and MELD is 500. For the RFR model the optimum number of trees for MELD is 100 and for APRI and MELD is 10.

**Figure 2:**
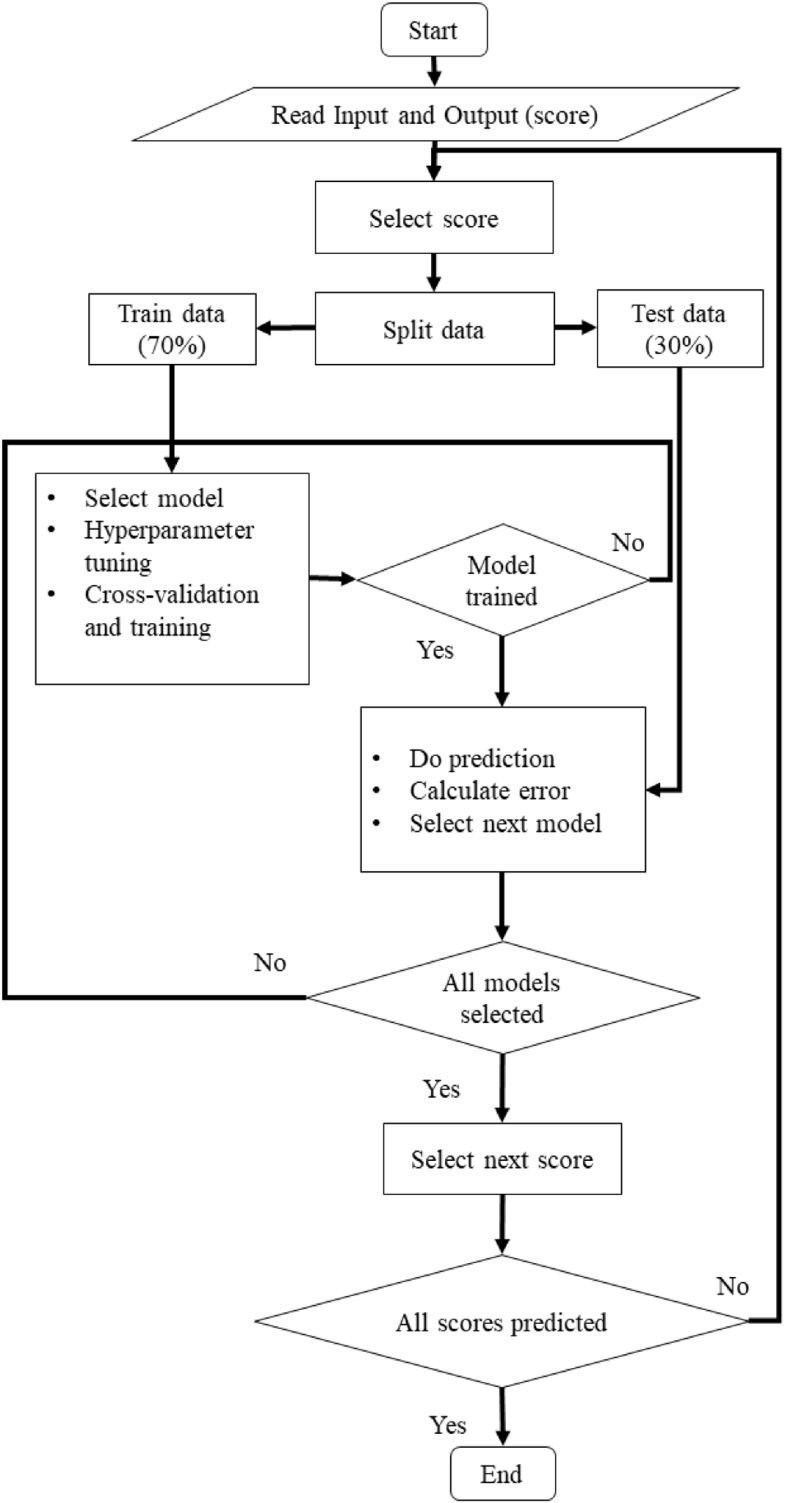
Flow chart of machine learning model.


Tenfold cross-validation is performed on the training dataset and the mean and standard deviation value of chosen error metrics are calculated. After the model is trained and cross-validated, the test dataset is used to predict the scores. The performance of regression analysis is evaluated based on mean absolute error (MAE) and root mean squared error (RMSE). The conceptual algorithm and flowchart (Fig. 2) describe the data analysis process.

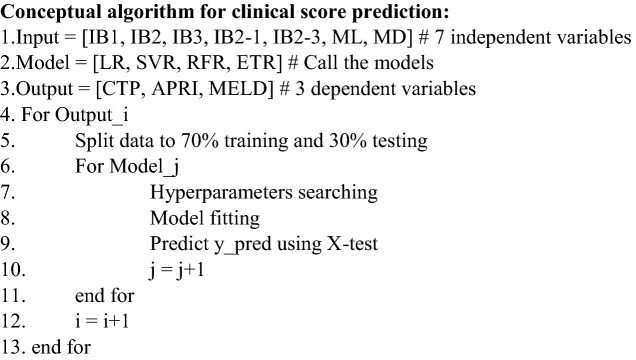


Usually, the predicted score using different regression methods gives a floating number. The nomenclature of medical scores (CTP and MELD) is the whole number. APRI score is one digit after the decimal. For this reason, the predicted scores are rounded off to nearby whole numbers in the case of CTP and MELD. The predicted APRI score is rounded to one digit after the decimal.

In this work, any feature selection and feature importance techniques are not adapted. Still other than the common error metrics, adjusted R^2^ value and Akaike information criterion (AIC) are also calculated for all the models. The adjusted R^2^ value increases if the selection of independent variables improves the model. To choose the best feature combination AIC value was calculated. The minimum AIC represents the best model or predictors combination. The formulas used for the calculation of the adjusted R^2^ and the AIC values are shown in Section 4 of the supplementary material.

## Results

**Figure 3:**
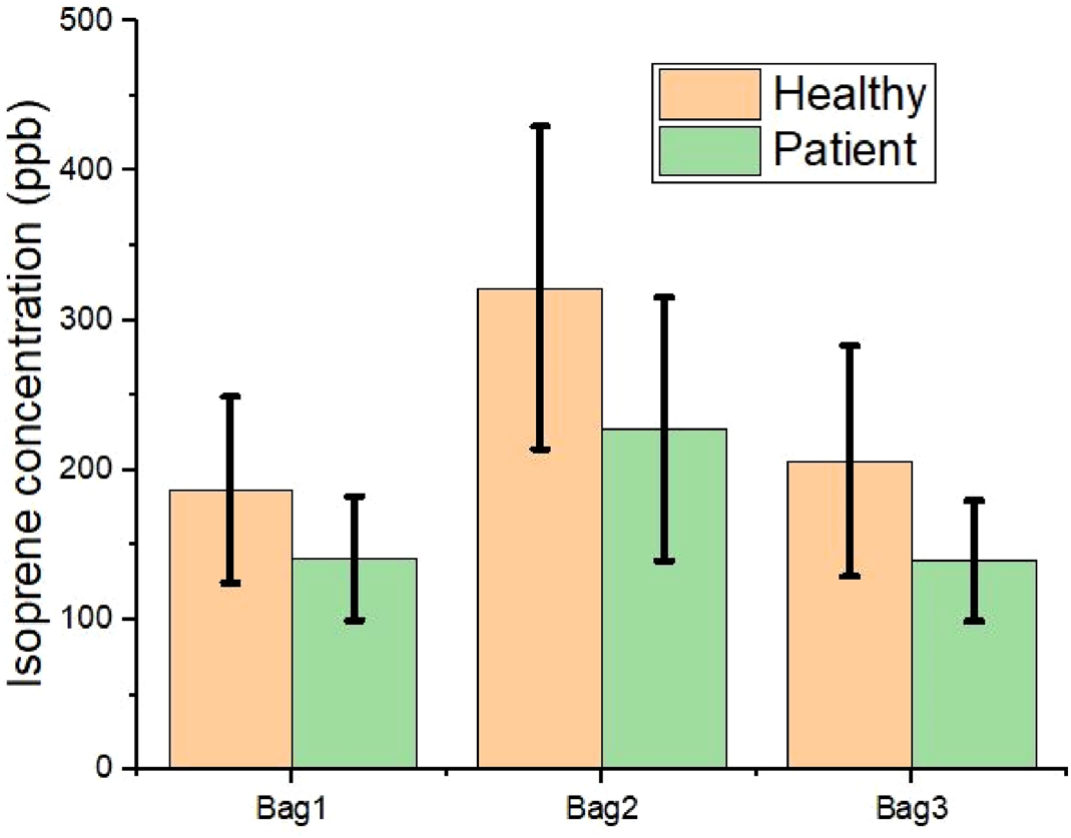
Isoprene concentration (ppb) from collected exhaled breath of patient and healthy group (Bag1: before physical activity, bag2: After physical activity, bag3: after 15 min of rest).

Figure 3 shows the mean and standard deviation (SD) plot of isoprene concentration from three different bags from the healthy and patient groups. As said earlier the range of isoprene is wider and overlap between the two groups. To observe the isoprene behavior in the said groups the trend change in the concentration profile is the priority of this work.

**Figure 4:**
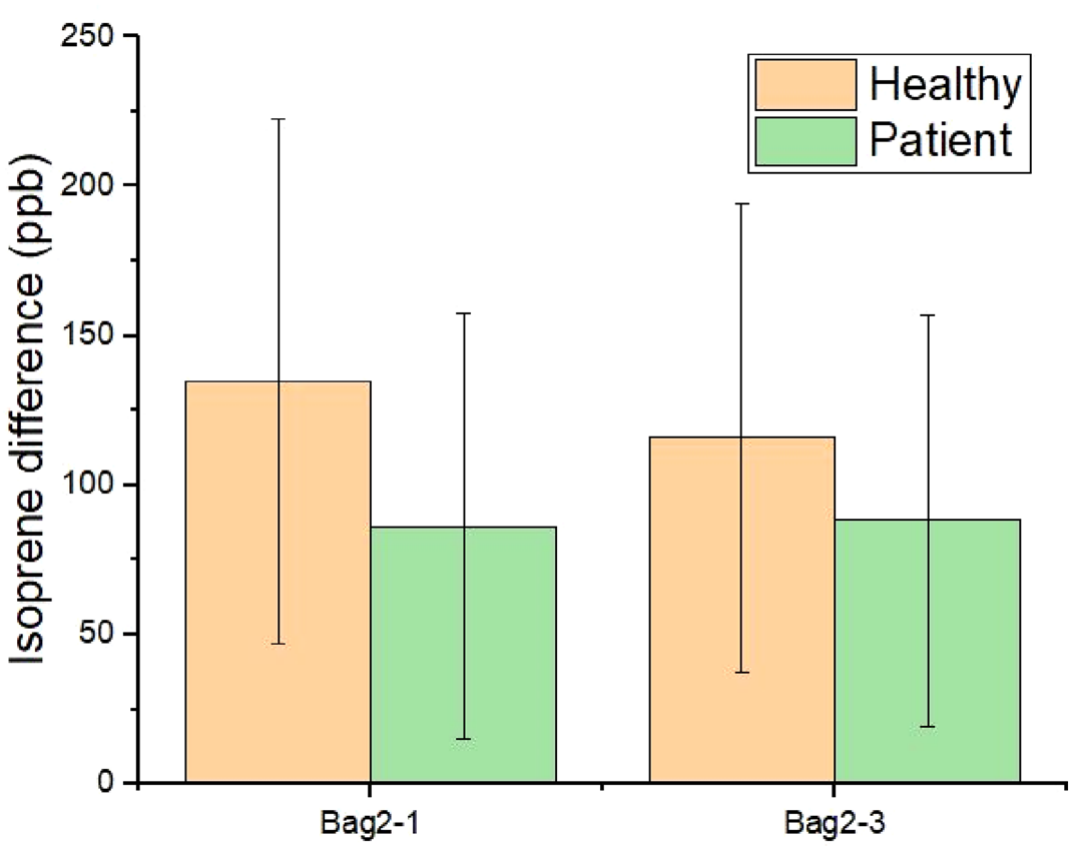
Isoprene concentration (ppb) difference from collected exhaled breath of patient and healthy group (Bag2-1: before physical activity and after physical activity, bag2-3: after physical activity and after 15 min of rest).

**Figure 5:**
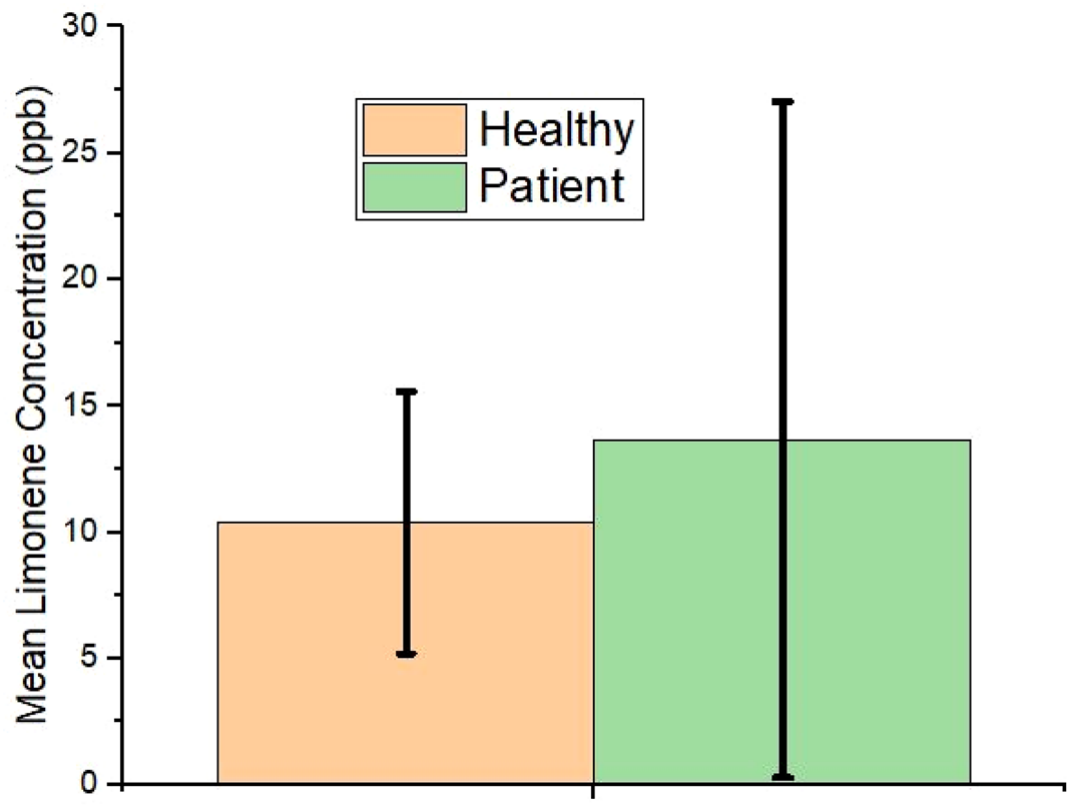
Mean limonene concentration (ppb) difference from collected exhaled breath of patient and healthy group.

**Figure 6:**
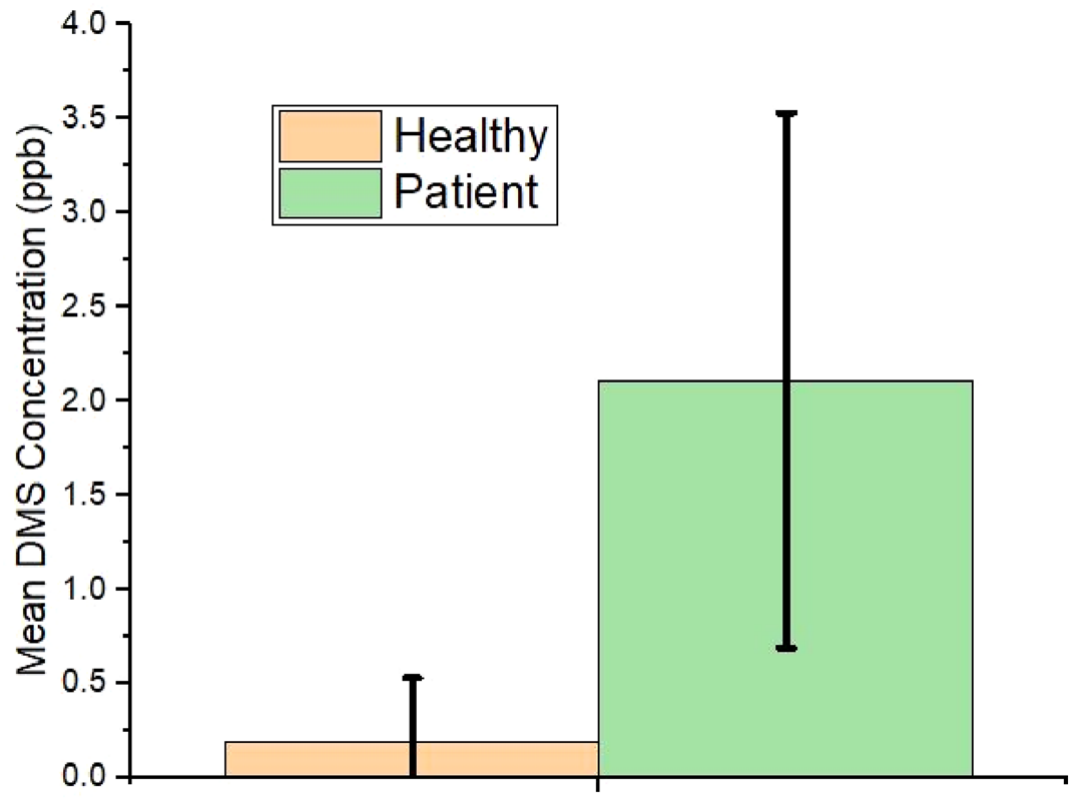
Mean DMS concentration (ppb) difference from collected exhaled breath of patient and healthy group.

The concentration of isoprene difference before and after a workout is shown in Fig. 4. The mean limonene concentration for all subjects in bag1, bag2, and bag3 was found as 15 ppb, 14.92 ppb, and 13.53 ppb. As per the designed breath collection protocol, the concentration of limonene remains almost the same in the three bags, but the mean concentration of limonene is different in the patient group and the healthy group as shown in Fig. 5. Similarly, the mean DMS concentration in the three bags are 1.0, 1.05, and 0.69 ppb but the mean concentration of DMS in the patient group and the healthy group (Fig. 6) are 2.2 ppb and 0.2 ppb, respectively.

### Statistical analysis

An independent one-tailed t-test conducted on the target VOCs concentration data collected from both the groups and a dependent one-tailed t-test performed on the isoprene concentration before and after performing physical activity on the total study subjects. To find the mean concentration of biomarkers either more or less between the groups is one-directional so a one-tailed t-test is adapted^39^. An independent t-test is performed to observe the existence of a significant difference between patients (n = 17) and the healthy group (n = 28) with a significance level = 0.01. The mean concentration of isoprene from each of the three bags is tested including the difference in isoprene concentration before and after performing a physical activity with mean limonene and mean DMS concentration between two groups. A dependent t-test is performed on the entire population between bag1 and bag2 that indicates the possibility of a significant difference in Isoprene concentration after physical activity. Similarly, another dependent t-test is performed on the entire population between bag2 and bag3 that indicates the possibility of a significant difference in isoprene concentration after rest.

Table 2 explains that the target VOCs shows significantly different concentration profile in both the groups and the isoprene compound which is a by-product of cholesterol synthesis shows a trend change in the concentration.

**Table 2:** Breath test parameters and t-test result.

Type of test	Parameter	*t* value	*p* value
Independent t-test on patients (n = 17) and healthy group (n = 28)	Isoprene_bag1	2.691	0.005
	Isoprene_bag2	3.042	0.002
	Isoprene_bag3	3.287	0.001
	Mean limonene	− 1.165	0.125
	Mean DMS	− 6.857	0.00001
Dependent t-test on total study subjects (n = 45)	Isoprene concentration before and after workout (bag1 and bag2)	9.239	0.00001
	Isoprene concentration after workout and rest (bag2 and bag3)	− 9.238	0.00001

### Regression result

Table 3 shows the error metrics for the test set and validation set of said regression analysis methods for the CTP, APRI, and MELD scores prediction.

**Table 3:** Test and cross-validation results for the prediction of CTP, APRI, and MELD scores.

		Validation result		Test result	
		MAE (Mean and SD)	RMSE (Mean and SD)	MAE	RMSE
CTP	ETR	0.143 ± 0.105	0.405 ± 0.357	0.071	0.267
	RFR	0.275 ± 0.261	0.437 ± 0.435	0.285	0.534
	SVR	0.368 ± 0.354	0.563 ± 0.616	0.285	0.654
	LR	0.489 ± 0.326	0.681 ± 0.566	0.362	0.557
APRI	ETR	0.206 ± 0.114	0.418 ± 0.341	0.143	0.249
	RFR	0.29 ± 0.186	0.407 ± 0.333	0.288	0.43
	SVR	0.442 ± 0.243	0.553 ± 0.365	0.332	0.429
	LR	0.54 ± 0.353	0.673 ± 0.471	0.258	0.339
MELD	ETR	0.94 ± 0.535	1.199 ± 0.673	0.785	1.101
	RFR	0.92 ± 0.626	1.055 ± 0.677	0.857	1.195
	SVR	1.256 ± 0.839	1.426 ± 0.893	1.071	1.488
	LR	1.104 ± 0.764	1.209 ± 0.77	1.341	1.626

**Figure 7:**
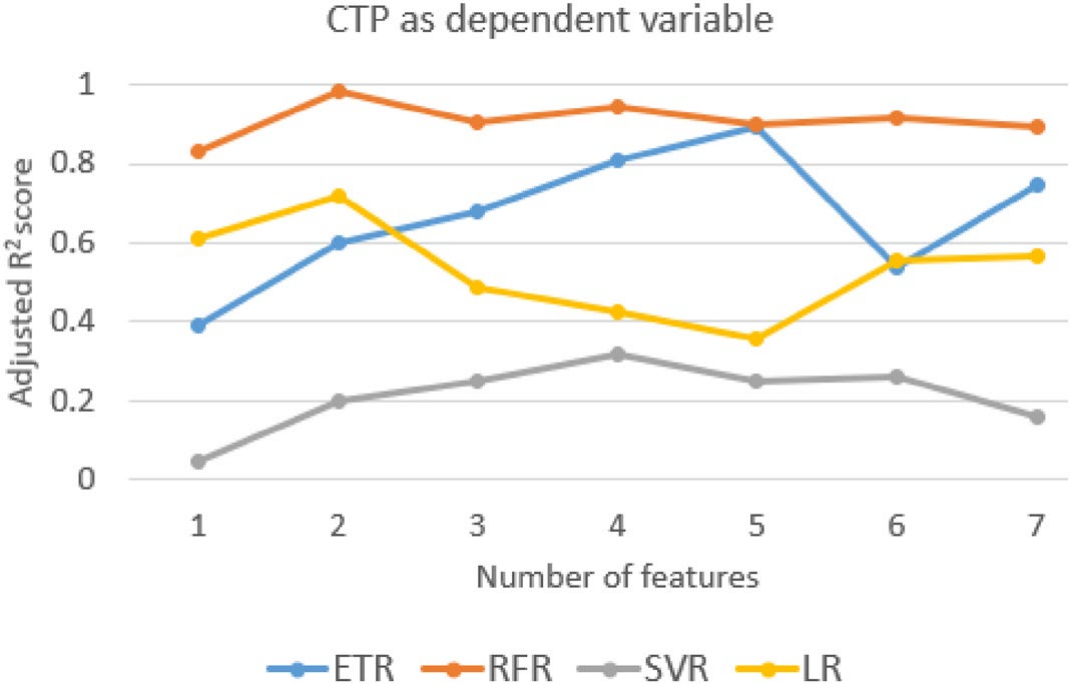
The adjusted R^2^ value for different sets of features with CTP score as the dependent variable.

**Figure 8:**
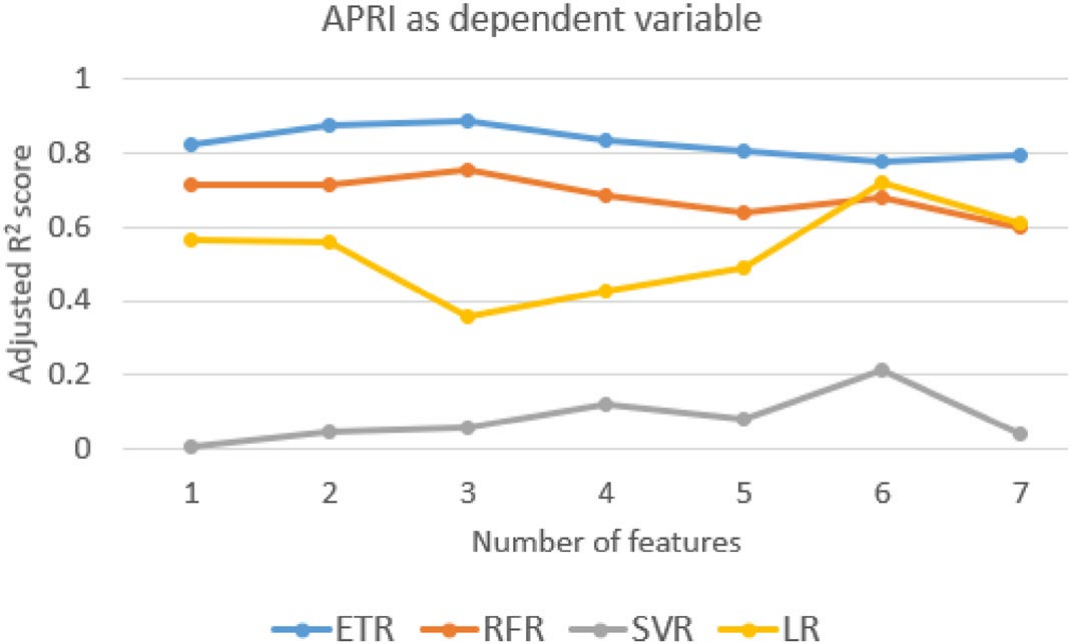
The adjusted R^2^ value for different sets of features with APRI score as the dependent variable.

**Figure 9:**
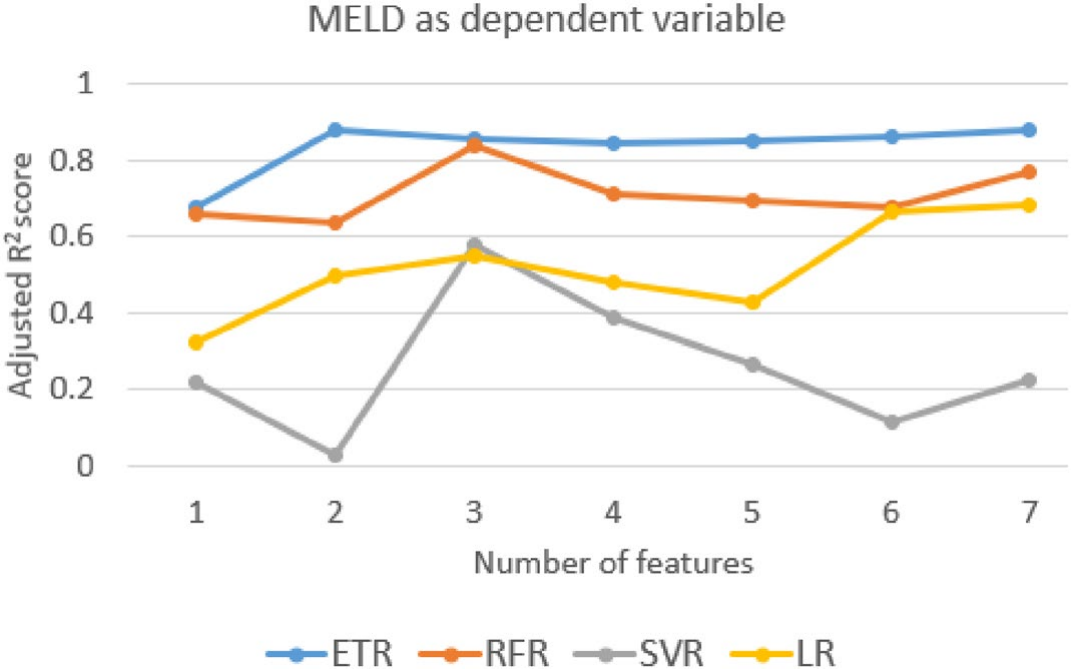
The adjusted R^2^ value for different sets of features with MELD score as the dependent variable.

The adjusted R^2^ value for different independent variable combinations is shown in Figs. 7, 8 and 9 and the selected independent variables set is shown in Table 4. The top five AIC values with the respective feature set are shown in supplementary Table S3. The formulas used for calculation are shown in supplementary section 4.

**Table 4:** **S**et of features used for adjusted R^2^ value calculation.

Number of features	Set of features
1	IB1
2	IB1 and IB2
3	IB1, IB2 and IB3
4	IB1, IB2, IB3 and IB2-1
5	IB1, IB2, IB3, IB2-1 and IB2-3
6	IB1, IB2, IB3, IB2-1, IB2-3 and ML
7	IB1, IB2, IB3, IB2-1, IB2-3, ML and MD

**Figure 10:**
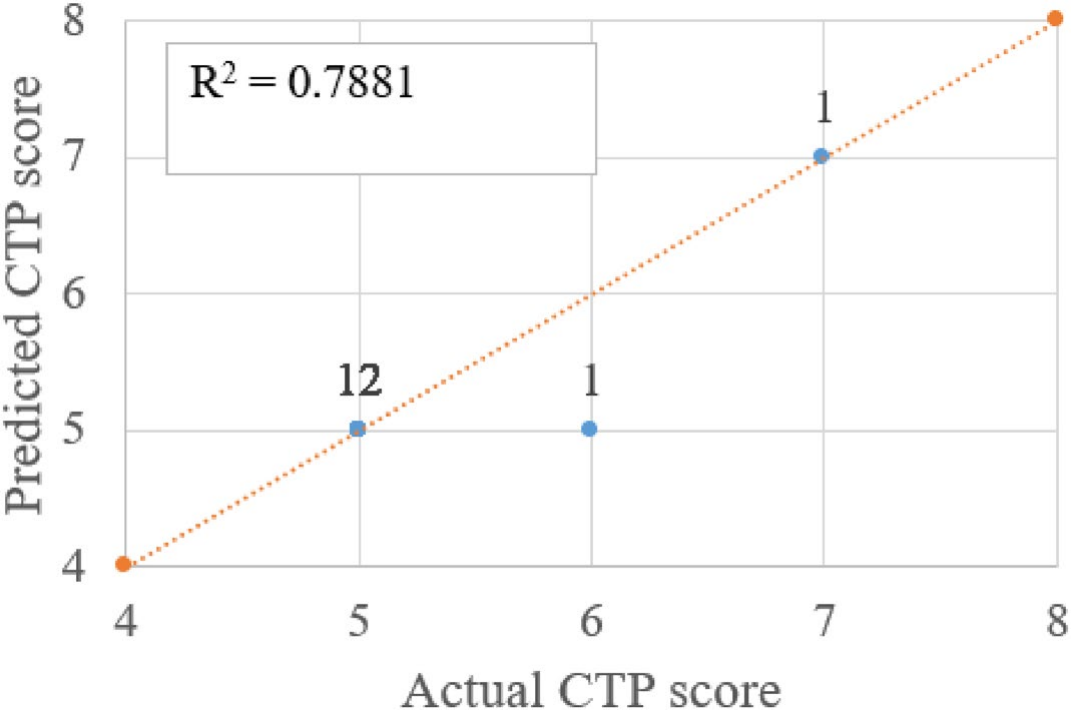
Comparison of actual and predicted CTP score (using ETR) and number of instances shown as label.

**Figure 11:**
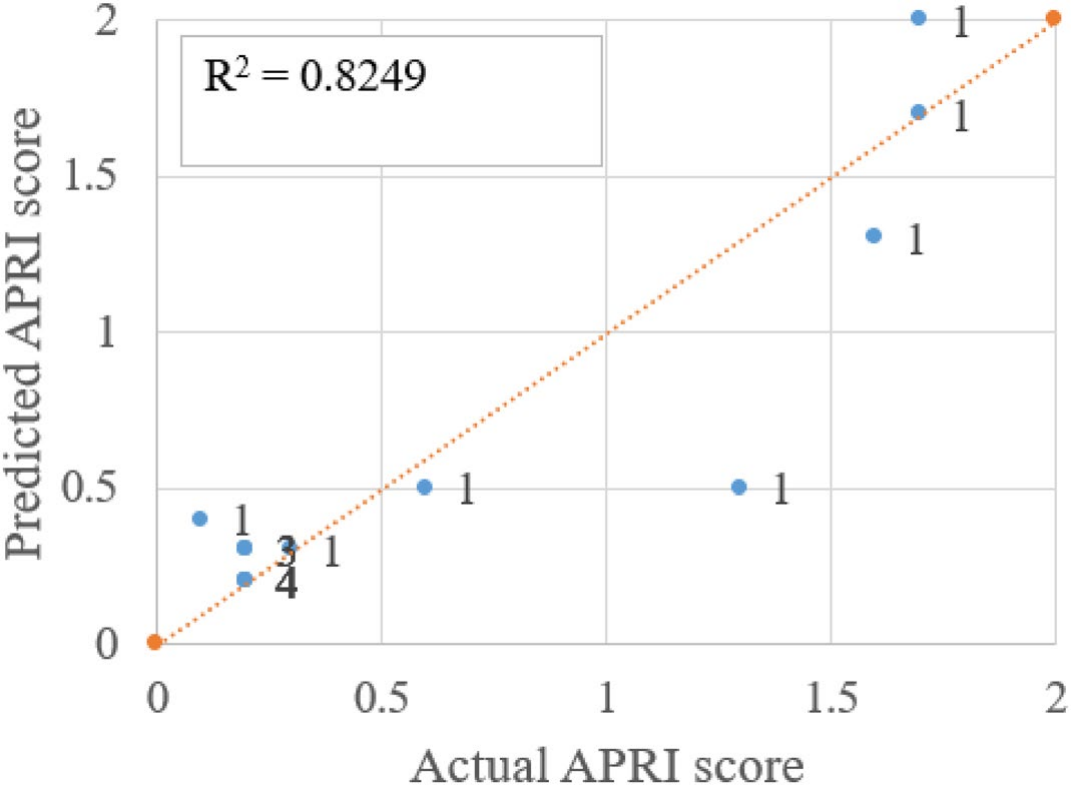
Comparison of actual and predicted APRI score (using ETR) and number of instances shown as label.

**Figure 12:**
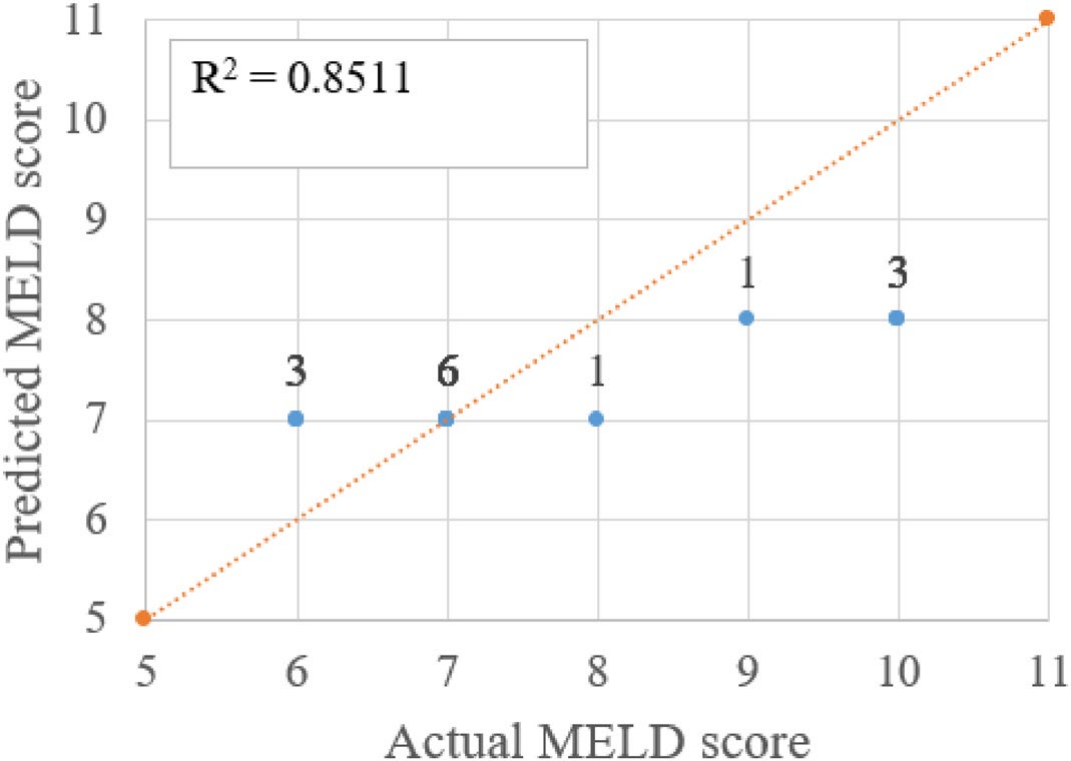
Comparison of actual and predicted MELD score (using ETR) and number of instances shown as label.

Figures 10, 11 and 12 shows a scatter plot between predicted score and real score with the counts or number of test subjects (digit shown next to marker) for overlapped entries. As the dataset distribution shows CTP score 5 is more in number. All healthy people and patients with liver disease at minor stage their CTP score is 5 which is the minimum value. The count number shows the entries in that coordinates. R^2^ value is more than 0.78. As the CTP score states that 5 to 6 score is considered as type-A and 7 to 9 as type-B. The above plot shows that there is one misclassification where the actual score is 6 and the predicted score is 5. If a CTP score of 5 got misclassified to 7 then a type A patient will be predicted as type B that is a serious misjudgment. The number of wrong predictions can be improved with an even distribution covering all possible score values. Figure 12 shows all the said regression method R^2^ values for three different scores.

## Discussion

The primary goal of this research is to evaluate the capability of the selected breath biomarkers analysis as a non-invasive method for monitoring liver disease by quantifying the up-and-down-regulated VOCs concentration in the breath sample as a result of the disease. A study protocol that involves a brief physical exercise to make a trend change in the VOCs concentration is a unique approach in this work. Three VOCs considered in this work are isoprene, limonene, and DMS. As we hypothesized a significant difference in the isoprene concentration of the biomarker is observed between the healthy group and the patient group after performing the exercise. This work is further extended by using the biomarker profile and machine learning regression methods, whereby liver-related clinical scores are predicted.

A significant difference was observed, as shown in Table 2. An ill liver is deprived of its efficient metabolism potential, mostly due to damage to active liver cells. Changes in metabolism alter the concentration of VOCs. The concentration of isoprene decreases as the liver fibrosis stage increases towards critical condition^19^. From previous studies, as described, the concentration window of isoprene is broad (varies from 50 to 500 ppb)^40^. To make better predictions or decisions using the machine learning approach, more independent variables are always appreciated. Rather than the absolute concentrations, this study considers the change in isoprene concentration as an important parameter and includes it in the dataset.

Figure 3 shows the isoprene concentration profile from the three breath samples from both groups. The isoprene concentration at rest (bag 1) and after rest (bag 3) in the liver group is lower compared to the healthy group. Isoprene concentration changes in human breath after exercise. In a study, it has been found that isoprene concentration is higher when the heart rate increases after a workout^30^. Also, we observed the same in our study. In Table 2, a dependent t-test shows that before and after exercise, the isoprene concentration is different for all study subjects. Adenosine triphosphate (ATP) is the primary high-energy phosphate particle that empowers muscle compression. In the ATP production process, this acetyl-CoA enters the Krebs cycle to drive oxidative phosphorylation. This procedure mainly happens in mitochondria, which is the major source of ATP. In this procedure, acetyl-CoA is produced which triggers the mevalonate cycle which is responsible for the production of isoprene in the human body^41,42^. The severity of the liver disease builds a hepatic accumulation, increases HMG CoA reductase (HMGCR) which results in dysregulation of cholesterol metabolism and affects the isoprene production^43–46^. The isoprene concentration profile gets affected because of the malfunction of the liver. After performing the exercise, the collected sample (bag 2) isoprene concentration is about two times higher than the bag 1 concentration, but in the case of a liver patient, the amount it increases is lower in comparison to the healthy group.

The mean concentration of limonene, which usually does not increase after performing the exercise, remains nearly the same in all three bags, so the mean limonene concentration from the samples of both groups is considered. The mean limonene concentration of the healthy group is less compared to the liver patient group as shown in Fig. 5. Limonene is common in healthy people's and liver patients' breath because it is commonly found in food and beverages that give citrus fruit flavor, and this is hard to avoid in the regular diet^27^. Limonene is obvious to appear in the exhaled breath of both the healthy and the patient groups, but the concentration remains higher in the latter group, and this was even proved in a recent study^47^. The drugs and nutrients metabolizing P450 enzymes were found to be deprived of the required quantity when the liver disease is in progression. In particular, CYP2C19 and CYP2C9 were down-regulated in production, and incomplete digestion of limonene appeared in the breath in a higher concentration^26^. To avoid any residuals of limonene, a minimum of eight hours of fasting and rinsing of the mouth with water just before the test is compulsorily imposed on all study participants.

The same trend was also observed with DMS concentration in the breath. As shown in Table 2, the DMS concentration in the breath is supposed to be higher in liver patients than in healthy people. We found the mean DMS concentration in patients higher than the healthy group (Fig. 6) and this type of result is also observed by other authors^25^. There is a possibility that a liver with a progressing stage of dysfunction may cause a change in the concentration of exhaled sulfur compounds^22,48^. Liver patients' exhaled breath carries a higher amount of DMS than healthy individuals.

Three datasets were designed, each comprised of seven input features and the predicted output variable for each dataset is CTP score, APRI score, and MELD score, respectively. Two different errors were used to evaluate each regression method. Four different regression methods were adapted. Comparison of the regression method is not the objective of this study. Ensemble learning (ETR and RFR) based ETR did a better prediction of scores compared to the other three said regression methods, especially SVR and LR. The interpretation of regression results based on the chosen error matrix (MAE and RSME) is described in the following paragraph for the said scores. As shown in Table 3, the mean value of CTP in the dataset is 5.5, and using ETR, the least MAE and RMSE are found to be 0.071 and 0.267, respectively, which makes up the MAE less than 5% of the mean CTP score. Table 3 shows the APRI score prediction. The minimum MAE and RMSE are 0.143 and 0.249, respectively. The mean APRI score is 0.5 and the errors are more than 28% of this value. For the MELD score, the mean value is 7.5. The MAE and RMSE are found to be 0.785 and 1.101, respectively, as shown in Table 3. The error is less than 15% of the mean MELD value of the dataset.

A mixed variation is observed in the adjusted R^2^ value for all the combinations of models and independent variables (Figs. 7, 8 and 9). The independent variables were added in a general way as shown in Table 4. With the addition of IB2 with IB1 for most of the models, there is an improvement in the adjusted R^2^ value. This can be interpreted as IB2 having a good contribution to the model predicting power. For the CTP score, the adjusted R^2^ value for the chosen feature set is 0.891 and the adjusted R^2^ value for the best model feature combination is 0.982. The difference in the adjusted R^2^ value between the chosen feature set and the best model feature combination is 0.091. This may be considered as a small difference and the considered feature set seem to be acceptable based on the adjusted R^2^ value. For the APRI score and the MELD score, the difference in the adjusted R^2^ values is 0.08 and 0.01, respectively. From the dataset and the model, AIC values are also calculated and the top five values are shown in supplementary Tables S3–S5. Only the linear regression model was used with MSE as the maximum likelihood function. The difference in score between the best model and the proposed model is 0.064 for the CTP score, 2.081 for the APRI score, and 0.308 for the MELD score.

**Figure 13:**
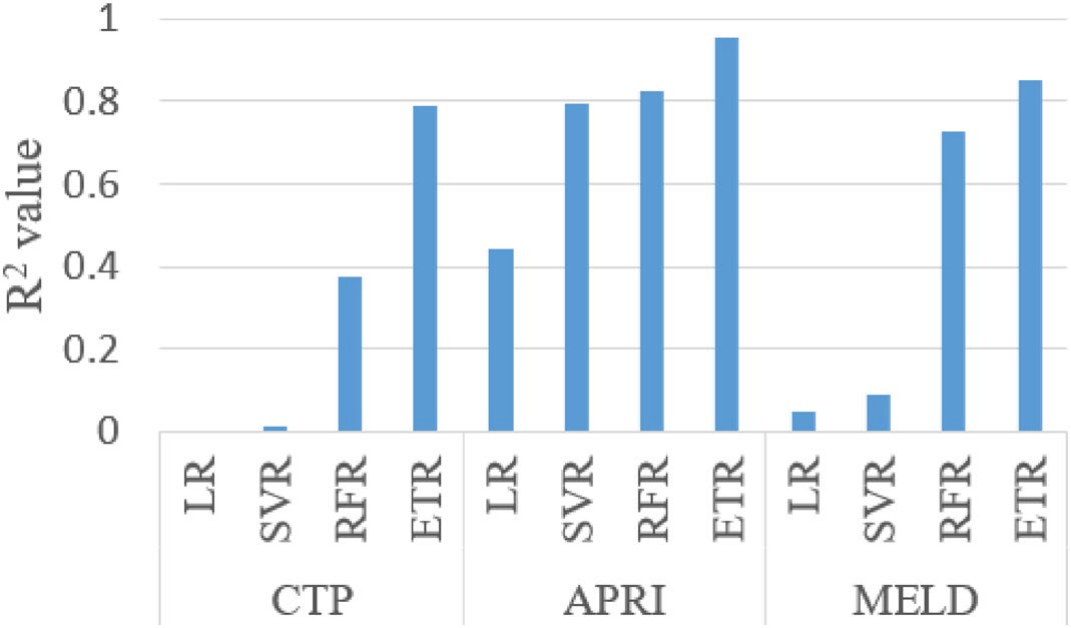
R^2^ value of actual score to predicted score using four different regression method for CTP, APRI and MELD score.

There is one misclassification and the R^2^ value is 0.78 between real and predicted CTP score plotted in Fig. 10. The APRI score states that if the value is less than 0.5 there is no fibrosis present and more than 1.5% strong chance of having fibrosis. Three misclassifications in APRI score prediction with R^2^ value between the actual and predicted score is 0.82, as shown in Fig. 11. Three misclassifications are observed where the real value is 10 and the predicted score is 8. Figure 12 shows the R^2^ value of 0.85 between the real and predicted score. Figure 13 puts together the performance of four different regression methods for predicting the scores. The ensemble learning fits very well with this dataset and predicts scores very close to the real scores.

## Conclusion

Our pilot study demonstrated that Isoprene, limonene and DMS can be potential biomarkers for liver disease. This study design involves an exercise in the breath collection protocol that includes healthy subjects and liver patients. There is a significant difference in the breath profile that has been found between liver patients and healthy people. Breath profile data is analyzed using four different regression methods and advantages of proposed method are highlighted quantitatively. We showed that clinical scores can be predicated with our machine learning regression approach and breath profile data. The regression result can estimate the clinical scores which imply the concentration of biomarkers varies according to the liver condition. The designed breath collection protocol includes a brief duration exercise which is a novel approach according to the best of our knowledge in the field of liver-related study. By training our model with big dataset, a non-invasive early disease diagnosis and prognosis.

## Supplementary Information

Supplementary Information.
