# Supplementary material for: A pilot study for the prediction of liver function related scores using breath biomarkers and machine learning
Source: Sci Rep. 2022 Feb 7;12:2032. doi: 10.1038/s41598-022-05808-5 (PMC8821604; doi:10.1038/s41598-022-05808-5)
Supplement: Supplementary file 1 — Supplementary Information. [file 41598_2022_5808_MOESM1_ESM.docx]

**A PILOT STUDY FOR THE PREDICTION OF LIVER FUNCTION RELATED SCORES USING BREATH BIOMARKERS AND MACHINE LEARNING**

**Supplementary information**

**Section1: Clinical score calculation**

The formula for the APRI score is [(AST/upper limit of the normal AST range) X 100]/Platelet Count. First, divide AST count by the upper limit of the normal AST range. Most experts say that 40 is a good value to use here. Then multiply that answer by 100 and at last divide that answer by your platelet count. If the score is less than 0.5 it is free from fibrosis and if it is higher than 1.5 then the liver has scaring and leads to cirrhosis^1^.

Following table shows five clinical parameters and there is a different range accordingly it score some values finally it gets totaled to deliver the CTP score^2^.

Supplementary Table 1. CTP score calculation

| TBIL | <2mg/dL (<34.2 umol/L) | +1 |
| --- | --- | --- |
|  | 2-3mg/dL (<34.2-51.3 umol/L) | +2 |
|  | >3mg/dL (>51.3 umol/L) | +3 |
| ALB | >3.5g/dL (>35g/L) | +1 |
|  | 2.8-3.5 g/dL (28-35 g/L) | +2 |
|  | <2.8 g/dL (<28 g/L) | +3 |
| INR | <1.7 | +1 |
|  | 1.7-2.2 | +2 |
|  | >2.2 | +3 |
| ASC | Absent | +1 |
|  | Slight | +2 |
|  | Moderate | +3 |
| HE | No | +1 |
|  | Grade 1-2 | +2 |
|  | Grade 3-4 | +3 |

CTP score is between 5 and 6 treated as class A, between 7 to 9 is class B and above 10 is class C. Class A, B and C has one year of survival 100%, 80% and 45%, respectively.

End stage liver health can be judged using MELD score. MELD score calculated counting on TBIL, CRE, INR, Na and dialysis in last two weeks^2^. Renal dysfunction because of kidney failure may disturb the MELD score prediction.

MELD=10* (0.957* ln [Creatinine]) + (0.378* ln [Bilirubin]) + (1.12 ln [INR])) + 6.43

And MELD-Na=MELD + 1.32 x (137 - Na) - [0.033 x MELD*(137 - Na)].

Interpretation of MELD score is less than 9, 1.9% mortality. As the score increase mortality percentage also increases.

**Section2: Clinical score distribution**


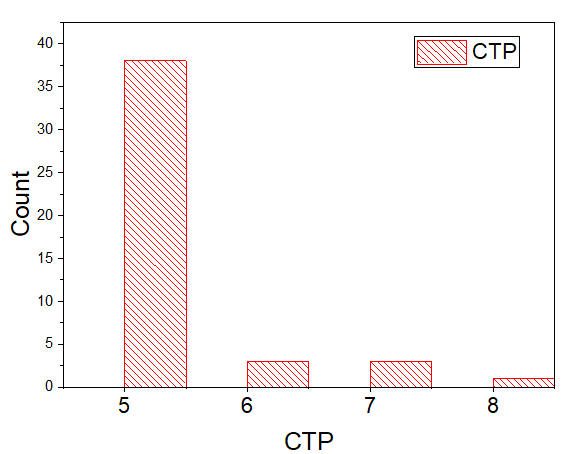


**Supplementary figure 1.** Distribution of CTP score among study subjects


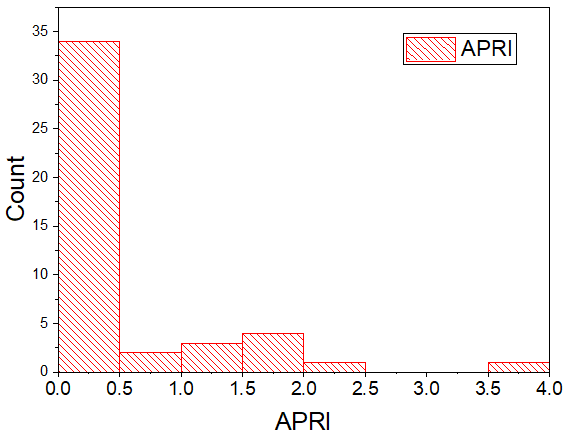


**Supplementary figure 2.** Distribution of APRI score among study subjects


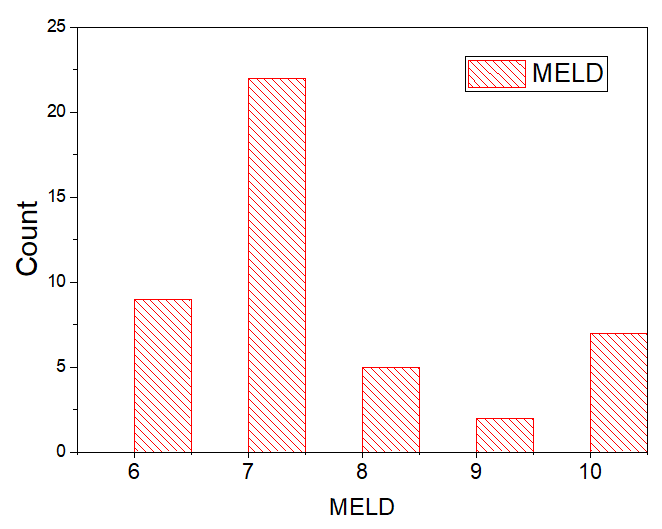


**Supplementary figure 3.** Distribution of MELD score among study subjects

**Section3: Hyperparameters of regression methods**

**Supplementary table 2.** List of major parameters of chosen regression methods

| **SVR** | **Parameters** | **CTP** | **APRI** | **MELD** |
| --- | --- | --- | --- | --- |
|  | **C** | 0.01 | 1 | 0.01 |
|  | **Kernel** | Linear | Linear | Linear |
|  | **Degree** | 0 | 0 | 0 |
|  | **Negative mean absolute error** | -0.32 | -0.31 | -0.88 |
| **RFR** | **Number of trees** | 10 | 10 | 100 |
|  | **Maximum depth** | 5 | 3 | 1 |
|  | **Maximum features** | 7 | 7 | 7 |
|  | **Minimum split size** | 3 | 5 | 2 |
|  | **Negative mean absolute error** | -0.2 | -0.27 | -0.89 |
| **ETR** | **Number of tress** | 10 | 500 | 500 |
|  | **Maximum depth** | 5 | 10 | 5 |
|  | **Maximum features** | 7 | 7 | 7 |
|  | **Minimum split size** | 2 | 3 | 2 |
|  | **Negative mean absolute error** | -0.15 | -0.24 | -0.85 |

**Section4: Calculation of adjusted R^2^ and Akaike information criterion (AIC)**

$$Adjusted R^{2}=1-\frac{\left( \left( 1-R^{2} \right)\left( N-1 \right) \right)}{N-p-1}$$

Where, R^2^ = sample R-square

N = Sample size = Test size sample is 14 (30% of the dataset)

P = Number of predictors = 7 features as described in the dataset

The Akaike information criterion (AIC) is a mathematical method for evaluating how well a model fits the data^3^.

$$AIC=N*ln\frac{SSe}{N}+2k$$

Where, N: number of observations

K: Number of parameters

MSE: Mean squared error

SSe: Sum square of errors

**Section5: AIC score for different set of features**

**Supplementary table 3**: Set of features and AIC score for a linear regression

| **Dependent variable** | **Features** | | **AIC score with CTP score as the dependent variable** | |
| --- | --- | --- | --- | --- |
| CTP score | | IB1, IB2, IB3, IB2-1, ML and MD | | -64.192 |
|  |  | IB1, IB2, IB3, IB2-3, ML and MD | | -64.170 |
|  |  | IB1, IB2, IB3, IB2-1, IB2-3, ML and MD | | -64.128 |
|  |  | IB1, IB3, IB2-1, IB2-3, ML and MD | | -62.271 |
|  |  | IB1, IB2, IB2-1, IB2-3, ML and MD | | -61.518 |
| APRI score | | IB1, IB2, IB3, IB2-3, ML and MD | | -59.569 |
|  |  | IB1, IB2, IB3, IB2-1, ML and MD | | -59.463 |
|  |  | IB1, IB3, IB2-1, IB2-1, ML and MD | | -58.514 |
|  |  | IB1, IB2, IB3, IB2-1, IB2-3, ML and MD | | -57.488 |
|  |  | IB1, IB2, IB2-1, IB2-3, ML and MD | | -54.329 |
| MELD score | | IB1, IB3, IB2-1, IB2-3, ML and MD | | 27.966 |
|  |  | IB3, IB2-1 and ML | | 28.211 |
|  |  | IB1, IB2, IB3, IB2-1, IB2-3, ML and MD | | 28.274 |
|  |  | IB1, IB3 and ML | | 28.278 |
|  |  | IB3, IB2-3 and ML | | 28.351 |

1. Petersen, J. R. *et al.* Evaluation of the aspartate aminotransferase/platelet ratio index and enhanced liver fibrosis tests to detect significant fibrosis due to chronic hepatitis C. *J. Clin. Gastroenterol.* **48**, 370–376 (2014).

2. Cholongitas, E. *et al.* Systematic review: The model for end-stage liver disease--should it replace Child-Pugh’s classification for assessing prognosis in cirrhosis? *Aliment. Pharmacol. Ther.* **22**, 1079–1089 (2005).

3. Pawitan, Y. *In All Likelihood: Statistical Modelling and Inference Using Likelihood*. (OUP Oxford, 2013).
